# Pathogenetic Link of Cardiac Rupture and Left Ventricular Thrombus Following Acute Myocardial Infarction: A Joint Preclinical and Clinical Study

**DOI:** 10.3389/fcvm.2022.858720

**Published:** 2022-06-09

**Authors:** Shan Ma, Ling Bai, Ping Liu, Gang She, Xiu-Ling Deng, An-Qi Song, Xiao-Jun Du, Qun Lu

**Affiliations:** ^1^Department of Internal Medicine-Cardiovascular, Cardiovascular Hospital, The Second Affiliated Hospital of Xi’an Jiaotong University, Xi’an, China; ^2^Department of Internal Medicine-Cardiovascular, The First Affiliated Hospital of Xi’an Jiaotong University, Xi’an, China; ^3^Department of Physiology and Pathophysiology, School of Basic Medical Sciences, Xi’an Jiaotong University, Xi’an, China; ^4^Baker Heart and Diabetes Institute, Melbourne, VIC, Australia

**Keywords:** acute myocardial infarction, cardiac rupture, left ventricular thrombus, platelets, inflammation

## Abstract

**Background:**

Cardiac rupture (CR) and left ventricular thrombus (LVT) remain important complications of acute myocardial infarction (MI), and they are currently regarded as independent events. We explored the pathogenetic link between CR and LVT by investigating a murine model of MI with a high frequency of CR and in patients with acute MI.

**Methods:**

MI was induced in mice, the onset of CR was monitored, and the hearts of mice with or without fatal CR were histologically examined. Between 2015 and 2022, from patients admitted due to acute MI, the data of patients with CR or LVT were retrospectively collected and compared to uncomplicated patients (control).

**Results:**

A total of 75% of mice (*n* = 65) with MI developed CR 2–4 days after MI. A histological examination of CR hearts revealed the existence of platelet-rich intramural thrombi in the rupture tunnel, which was connected at the endocardial site to platelet-fibrin thrombi within an LVT. In CR or non-CR mouse hearts, LV blood clots often contained a portion of platelet-fibrin thrombi that adhered to the infarct wall. In non-CR hearts, sites of incomplete CR or erosion of the infarct wall were typically coated with platelet thrombi and dense inflammatory cells. Of 8,936 patients with acute MI, CR and LVT occurred in 102 (1.14%) and 130 (1.45%) patients, respectively, with three cases having both complications. CR accounted for 32.8% of in-hospital deaths. The majority of CR (95%) or LVT (63%, early LVT) occurred within 7 days. In comparison to the control or LVT-late groups, patients with CR or early LVT reported increased levels of cellular and biochemical markers for inflammation or cardiac injury.

**Conclusion:**

CR and LVT after MI are potentially linked in their pathogenesis. LVT occurring early after MI may be triggered by a thrombo-inflammatory response following wall rupture or endocardial erosion.

## Highlights

- We provided histopathological evidence for the co-existence of an intramural thrombus and LVT at the site of a wall rupture in all mice with fatal or non-fatal CR.
- We observed a similarity between patients with CR and early LVT in higher circulating levels of cellular and biochemical markers for inflammation and cardiac injury.
- Our findings suggest that the pathogenesis of the early onset of LVT is triggered, at least in part, by CR or wall erosion, which results in a thrombo-inflammatory response and the formation of LVT.

## Introduction

Over the last decades, there has been a significant decline in the mortality of patients with acute myocardial infarction (MI), which is attributable to the progress in therapies, including the routine use of percutaneous coronary intervention (PCI) and effective medications. Earlier studies showed that PCI therapy reduced the incidence of CR (1). Before 2,000, in-hospital and 30-day mortality rates were approximately 10–20% and 25–35%, (2–4) respectively, due to acute pump failure, fatal arrhythmias, or cardiac rupture (CR). Currently, these figures range between 3 and 8% (5–7).

Cardiac rupture and left ventricular (LV) thrombus (LVT) are two important complications of acute MI. CR may occur as early as day 1 to a few weeks after MI and is associated with a very high mortality rate (up to 70%) (4, 8–10). Subtypes of CR are classified according to the location of the rupture, which may involve the free wall (FWR), the ventricular septum (VSR), or the papillary muscles (PMR) (4). Early FWR (within 48 h) usually exhibits as a narrow slit or a regional erosion of the infarct wall, whereas subacute rupture (up to 2 weeks) is characterized by regional thinning and bulging of the infarct wall (8, 11, 12). While its incidence has reduced to 1–2% from the previous 5–10%, (8, 13, 14). CR continues to be one of the major causes of in-hospital mortality for patients with acute MI (14, 15). Between 1980 and 1990, LVT was detected in approximately 20–30% of patients with anterior/apical-localized MI (10, 16). Early studies indicated that several factors were associated with a high risk of LVT, including larger enzymatic infarct size, clinical evidence of pump failure, severe apical asynergy, increased LV volumes, and a lower ejection fraction, (10, 16) as well as a higher risk of embolic events, including acute ischemic stroke (17–20) and higher mortality rates (16, 21). The current literature indicates LVT incidence of 3–5% in patients with acute MI (22–24). The commonly accepted view on the mechanism of post-MI LVT is regional stasis of blood as a consequence of loss of a wall motion (10, 16). Intriguingly, there is a considerable overlay in the time window for CR and LVT after MI, mostly within the first week (4, 22) and even as early as 24 h, (21) and both complications share similar risk factors such as large infarct size.

The mouse with MI has been shown to be the only animal model with a frequent onset of CR (25). Numerous mouse studies, including ours, have shown that CR occurs within 2–6 days after MI and that factors such as a large infarct size and a severe inflammatory response after MI promote the onset of CR (25–27). In the mouse model, occlusion of the left coronary artery induces MI that involves the anterior-lateral-apical free wall, while the ventricular septum and papillary muscles are spared; thus, this model only simulates free-wall CR in human patients (25, 26). The presence of LVT has also been noticed in the murine MI model (28, 29). However, despite the fact that antiplatelet interventions effectively reduced the incidence of CR, the potential link between CR and LVT has not been explored previously (30).

In the current study, we tested the hypothesis that both CR and LVT are mechanistically related. Specifically, CR constitutes the histopathological basis for the formation of LVT, which is particularly true for the early-onset LVT in human patients. Considering the urgent nature of patients with CR and the limitations of generating pathological data collection from patients with CR or LVT, we conducted a joint preclinical and clinical study examining the histopathology of CR and LVT in the mouse MI model and retrospectively analyzing the clinical characteristics of patients with CR or LVT.

## Materials and Methods

### Preclinical Study: Animals, Surgery, Monitoring, and Autopsy

All experimental procedures were approved by the Xian Jiaotong University School of Medicine Research Ethics Committee and were in accordance with the United States NIH Guide for the Care and Use of Laboratory Animals. We used 3–4 month-old male 129sv mice, which, as we previously showed, exhibited a higher incidence of CR after MI than other strains (26, 28). Animals were kept in standard housing conditions with four animals per cage at 21°C in a facility with a 12/12 h light/dark cycle and were provided free access to water and standard chow. As we described previously, (26) open-chest surgery was performed to induce MI or sham surgery under isoflurane-inhalation anesthesia. The total loss of animals, due to surgical accidents (within 24 h) or failure in occluding major coronary arteries, was less than 10%. Operated animals were closely monitored for 7 days to observe the incidence of CR. As we described previously, (26) rupture death was ascertained by autopsy findings of hemopericardium and a large quantity of blood clot in the chest. Heart failure death was determined by the presence of pulmonary congestion (i.e., an increase in wet lung weight) and a large quantity of chest fluid. Infarct size was estimated in all animals by the LV surface method, as previously described (26).

### Preparation of Histological Heart Images and Histological Examination

Hearts were harvested from mice that had died of CR or had been freshly culled, washed in cold saline, fixed (4% buffered formaldehyde for 24 h), and paraffin-embedded. The heart was transversely cut, and sections (5 μm) were collected every 100–200 μm (depending on the regions of interest), and an average of 40 sections were harvested per heart. As we previously described, (28, 30) tissue sections were stained by the hematoxylin and eosin (H&E) method or by Carstair’s method using the commercial kit (GENMED, Shanghai, China) to identify platelets (purple/light color). The accuracy of identifying platelet thrombi by Carstair’s stain has been documented previously (28, 31). Images were acquired using Olympus Image Pro Plus6 (Media Cybergenetics). Histological images were independently read by two investigators (SM and X-JD), who later agreed on the final histological diagnosis for each heart examined.

### Retrospective Clinical Study: Patient Cohort

Patients who were admitted to the First Affiliated Hospital of Xi’an Jiaotong University from January 2015 to December 2020 with a confirmed diagnosis of acute MI were registered. The Ethical approval was obtained from the Ethics Committee of The First Affiliated Hospital of Xian Jiaotong University for the collection and use of clinical data in this study. According to ACC/AHA guidelines, (32) a diagnosis of acute MI was based on the elevation of cardiac enzyme biomarkers (CK-MB or troponin-T) along with at least one of the following criteria: (1) symptoms consistent with cardiac ischemia, (2) development of pathologic Q waves on electrocardiography, or (3) ST-segment elevation or depression on electrocardiography. Patients who met the diagnostic criteria for CR and LVT were included in a case group. Control patients were randomly selected from acute MI cases without CR or LVT. The average group size was determined to be approximately four times that of the CR and LVT groups. The exclusion criteria were patients with (1) CR or LVT who did not first visit our hospital after the onset of their current MI, (2) history of abnormal renal function (eGFR < 90 ml/min/1.73 m^2^) or chronic renal failure, (3) malignant tumor diseases, (4) serious infectious diseases, and (5) autoimmune diseases.

### Clinical Diagnosis of Cardiac Rupture and Left Ventricular Thrombus

The diagnosis of CR was based on clinical manifestations with confirmation by echocardiography using a Phillips iE33 ultrasound system (S5–1 probe) present in our ICU. Patients with CR experienced dyspnea, murmurs in the precordial area, and a drop in blood pressure. Ultrasound showed an interruption of the continuity of the interventricular septum, the papillary muscle, or the free wall. Hydropericardium was featured as a hypoechoic dark area surrounding the myocardium, and hemorrhagic effusion was confirmed by echo-guided pericardiocentesis. The diagnosis of LVT was based on at least two echocardiographic views, showing a delineated echo-dense mass adjacent to, but distinct from, the endocardium in a segment of the wall exhibiting abnormal motion. An aneurysm was considered when the following echocardiographic criteria were met: protuberance of the involved segment, well-defined demarcation of the infarct lesion, absence of trabeculation in the involved segment, and akinetic or dyskinetic motion.

### Collection of Clinical Data

Patient data were collected from the electronic patient record system of the First Affiliated Hospital of Xi’an Jiaotong University. Smoking cigarettes within 1 month of admission was defined as smoking. Alcohol consumption was defined as drinking ethanol > 15 mL ethanol per day in women and > 30 mL in men. Diagnosis of hypertension was defined as blood pressure ≥ 140/90 mmHg and/or the current use of antihypertensive medications. Diabetes was defined as plasma fasting; glucose was ≥ 7.0 mmol/L (or the 2 h postprandial glucose > 11.1 mmol/L) and/or the current use of antidiabetic medication. Regarding the current onset of MI, information included symptom-to-admission interval, symptom-to-CR or LVT interval, blood pressure, and heart rate at the time of admission, MI type by ECG, PCI treatment, infarct localization, and Killip classification assigned for the presence and severity of heart failure according to AHA/ACCA (32) and ESC guidelines (33). The presence of a ventricular aneurysm or pericardial effusion was determined by echocardiography conducted during the in-hospitalization period.

Venous blood was collected at admission into anticoagulant tubes to measure markers for inflammation or myocardial injury. The counts of white blood cells (WBC), neutrophils, lymphocytes, and monocytes were obtained using an automated cell counter (HST201, Sysmex, Japan). Cardiac injury markers were determined at the Clinical Biochemical Laboratories of the First Teaching Hospital using an AU640 Clinical Chemistry analyzer (Olympus Diagnostica, Hamburg, Germany), including aspartate transaminase (AST), lactate dehydrogenase (LDH), α-hydroxybutyrate dehydrogenase (HBDH), creatine kinase (CK, CK-MB), and high-sensitivity troponin T (hs-TnT). The high-sensitive C-reactive protein (hsCRP) was determined by a high-sensitivity particle-enhanced immunoturbidimetric method using BN II (Siemens, Germany) with a detection upper limit of 9.5 mg/L, and it is presented as a categorical variable. The measurements of fibrinogen, fibrinogen degradation products (FDP), and D-dimer were determined using the latex agglutination test (Sysmex CA-7000, Sysmex, Japan).

### Statistics

Statistical analyses were performed using SPSS version 25.0. The results are presented as frequency (%) for categorical variables, with differences between groups compared using the 2 × C Chi-square test. Normally distributed continuous variables were presented as mean ± SD, and ANOVA was used to analyze the differences between groups. For parameters that were not evenly distributed, results are presented as median [interquartile range (Q25, Q75)] and tested using the Kruskal–Wallis one-way ANOVA test for differences between groups. In our patient cohort, because the ratio of missing values was 1.8–9.1%, patients with missing data were ignored, and the univariate analysis was performed. *P* < 0.05 was defined as statistically significant.

## Results

### Time-Course of Survival and Incidence of Cardiac Rupture in Mice

**FIGURE 1:**
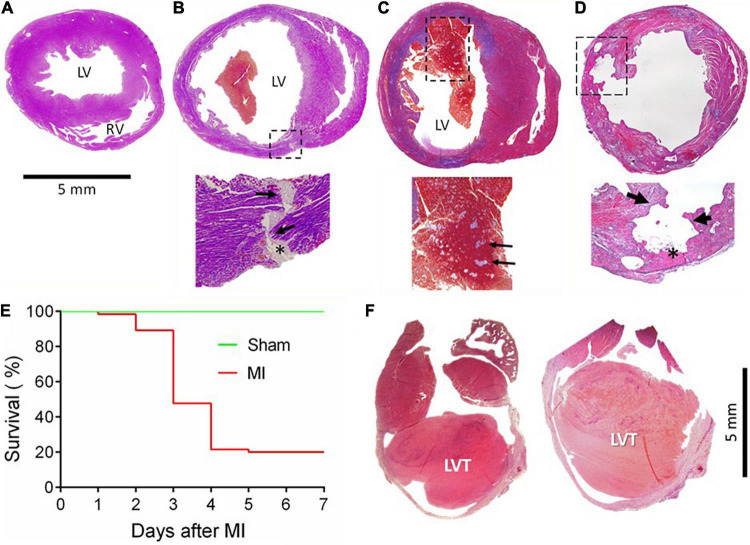
Gross cardiac histopathology of mice with acute or chronic MI and the post-acute MI survival curve. Images were stained by Carstair’s method in panels **(A–D)** and by H&E in F, showing hearts from a sham-operated mouse **(A)** or from a mouse with acute MI and developed CR **(B)** with a transmural rupture tunnel (indicated by arrows) containing platelet thrombi (*). **(C)** The heart of a mouse and died of acute heart failure after MI. Note that in the LV chamber, a large blood clot containing a mosaic of platelet-rich white thrombi, the structure suggesting that platelet thrombi were formed prior to death rather than developing postmortem. **(D)** Heart from a mouse killed on day 3 after MI with severe erosion of the infarct wall (indicated by broken lined square and arrows). The site of erosion was partially filled with platelet-rich thrombi (*) that was coated with dense immune cells. **(E)** Survival curves of mice with the sham operation (*n* = 15) or with acute MI (*n* = 65). Almost all fatalities within the first 2–4 days after MI were due to rupture. **(F)** The hearts of mice culled in week 4 after MI showed the presence of chronic left ventricular thrombus (LVT).

In the first batch of 65 mice with MI, animals were monitored for up to 7 days, and the cause of death was determined by autopsy. The infarct size, estimated from the surface of the LV, was 30–60%. The control group consisted of eight mice who received a sham operation and an additional five mice who had an infarct size of < 10%, which was defined as a failure to occlude a main coronary artery. Figures 1A–D shows a normal heart and common morphological findings of hearts from mice that died within the 7 days, including chamber dilatation and thinning of the infarct wall. Figure 1B shows the heart of a mouse that died of CR with the rupture site at the border zone and the transmural rupture tunnel blocked by platelet thrombi. In a mouse that died of heart failure, we observed the presence of a large and fresh LVT that contained mosaic structures of platelet thrombi (Figure 1C): such a morphological feature is consistent with the Lines of Zaha and rules out the possibility of the postmortem formation of a blood clot. Significant erosion of the infarct wall was observed in a mouse heart (Figure 1D). Figure 1E shows the Kaplan–Meier survival curve of the operated mice over the 7-day period. A total of 52 mice died (80%, 52/65). Of them, three died of heart failure (one on day one and two on day four), and the rest (*n* = 49) died of CR within 2–4 days after MI (75%, 49/65). The remaining 13 mice were followed for up to 28 days and then culled at the end. Of them, two mice had a large LVT upon autopsy and histology (Figure 1F).

### Histopathological Features in the Hearts of Mice That Died of Cardiac Rupture

**FIGURE 2:**
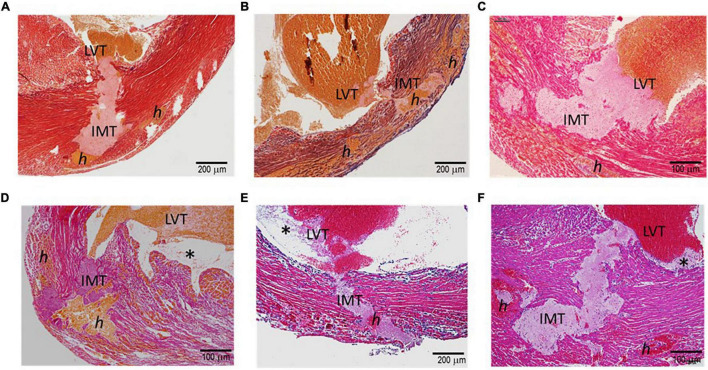
Histopathology of the hearts of mice with acute MI and developed fatal ventricular free-wall rupture. The images presented were from six hearts stained using the Carstair’s method **(A–D)** or H.E. method **(E,F)**. Transmural rupture with rupture tunnels containing intramural an (IMT), as well as intra-myocardial hemorrhage, indicated as “*h*,” was observed in all hearts. Furthermore, IMT was in close contact with LVT (LVT, all panels). Panels **(D–F)** show LVT embedded with platelet-rich thrombi adjacent to the infarct wall (*). Panels **(E,F)** show dense inflammatory cells at the interface of white thrombi of IMT and LVT.

Figure 2 displays histological features of the infarcted wall of the LV from mice that died of CR after MI. All mice had MI at the anterior-lateral-apical wall. Significant destruction of the LV wall, intra-myocardial hemorrhage, and dense inflammatory cell infiltration was evident in all hearts (Table 1). Also, identified in all hearts was a rupture tunnel, which was packed with platelet-rich intramural thrombi (IMT) (Table 1 and Figures 2A–F). Another common finding is the presence of thrombi in the LV chamber in close contact with IMT at the endocardial site of CR (Figure 2). Blood clots within the LV chamber usually contained a portion of platelet-fibrin thrombi adjacent to the infarct wall (Figures 2D–F).

**TABLE 1:** Summary of the histopathological findings of infarct hearts of mice that either died of the spontaneous onset of cardiac rupture (CR) or were freshly culled 3–5 after MI.

Histological findings	CR (*n* = 10)	Freshly culled (*n* = 14)
Severe inflammatory infiltration of the infarct wall	10	8 (57.1%)
Intramural hemorrhage and hematoma	10	9 (64.3%)
Disruption of necrotic cardiomyocytes	10	9 (64.3%)
Wall rupture (transmural or non-transmural)	10 (transmural)	6 (42.9%) (4 transmural, 2 wall erosion)
Intramural thrombus (IMT)	9	6 (42.9%)
IMT linked to LVT at the endocardial site of the rupture tunnel	10	3 (21.4%)
LV chamber blood clots containing platelet-fibrin-rich thrombi and inflammatory cells	7	6 (42.9%)

*IMT, intramural thrombus.*

### Histopathological Features of Infarct Hearts of the Mice That Had Been Freshly Culled

**FIGURE 3:**
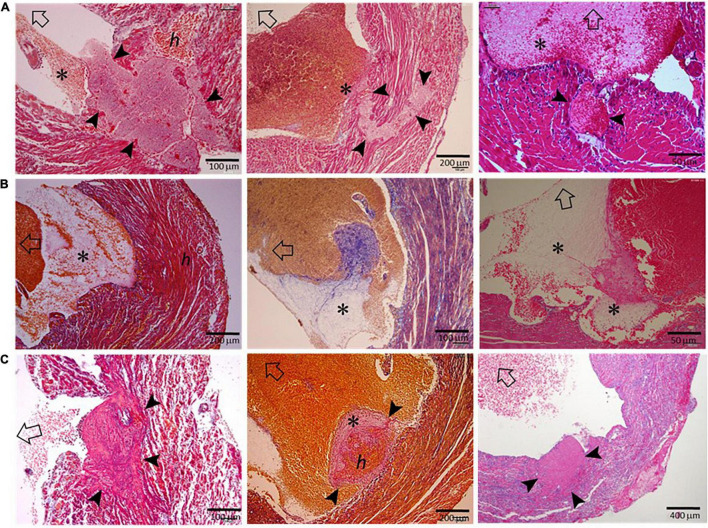
Histopathological findings of hearts from mice that had been freshly culled after MI. The images presented were from nine hearts of mice culled on days 3–5 after MI, and the sections were stained using either the Carstair’s method or the H&E method. Open arrows point in the direction of the LV chamber. **(A)** The presence of transmural or non-transmural rupture and intramural thrombi (arrowheads) that are closely linked with LVT (*). **(B)** The presence of a ventricular thrombus that contained platelet-fibrin portion (*) adjacent to the infarct wall. **(C)** Platelet-rich thrombi (arrowheads) either rooted in the erosive wall or stemmed from the infarct wall. Note the dense inflammatory cells surrounding the thrombus.

To exclude the potential effect on histopathology from postmortem changes, 14 mice with MI (estimated infarct size 39–55%) were culled 3–5 days after MI, and their hearts were harvested for histological analyses (Figure 3). Four mice died suddenly owing to stress associated with the anesthesia induction, and CR was confirmed by immediate autopsy. Similar to these hearts from mice that died of CR, all hearts from culled mice had significant infarct wall thinning and chamber dilatation. Intramural hemorrhage, disruption of necrotic myocardial fibers, and intense inflammation of the infarct wall were observed in 8–9 out of 14 hearts examined (Table 1). Transmural or nearly transmural CR was found in four hearts and an intramural thrombus in six hearts (Figure 3A). Importantly, six hearts (42.9%) displayed LV blood clots that contained a portion of platelet-fibrin-rich thrombi adjacent to the wall (Figure 3B). In two hearts, wall erosion was evident, with platelet thrombi covering a portion of the erosion (Figure 3C). Another two hearts had round-shaped platelet thrombi within the LV cavity that tightly adhered to the infarct wall and embedded dense inflammatory cells at its boundaries (Figure 3C).

### Histological Changes Leading to the Onset of Left Ventricular Thrombus

**FIGURE 4:**
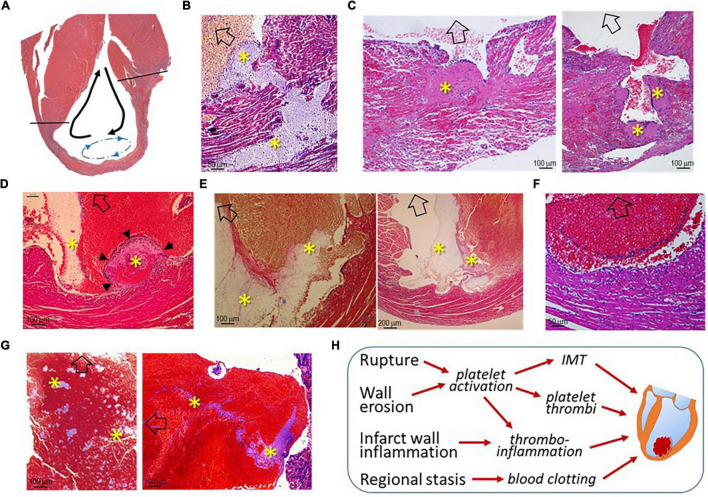
Histological images of infarct mouse hearts represent different pathological modes that ultimately result in LVT. All mouse hearts were collected 3–5 days after MI. In panels B-G, open arrows indicate the direction of the LV chamber. **(A)** Akinesis of the infarct segment with regional stasis (blue dashed arrow-circle line) that promotes the formation of LVT. The black arrows indicate the blood current in the LV chamber. **(B)** Extension of intramural thrombi (IMT) at the site of an endocardial tear to form LVT; **(C)** Infarct wall erosion with the formation of platelet thrombi (*) at the interface between infarct tissues and ventricular blood. The continuous enlargement of the thrombus would form LVT. **(D)** The early phase of LVT (arrowheads) stemmed from the infarct wall and was tightly adhered to a blood clot that contains a portion of platelet-fibrin thrombi (*) adjacent to the infarct wall. **(E)** Blood clots within the ventricular chamber containing platelet-fibrin thrombi (*) adjacent to the infarct wall. **(F)** Inflammatory infiltration at the surface of blood clots adjacent to the infarct wall. **(G)** Large ventricular thrombus embedded in a mosaic of platelet-rich thrombi (*). **(H)** The summary of histological modes that would lead to the formation of LVT after MI.

As illustrated in Figure 4A, LVT after MI has generally been regarded as a consequence of regional stasis due to poor wall movement, which facilitates the formation of blood clots. To better understand the pathogenesis of LVT after MI, we examined the hearts of infarct mice for histopathological signs of established or an early phase of the formation of LVT. We observed multiple histological modes related to LVT formation, including CR-associated IMT growing toward the LV chamber (Figure 4B) or growing platelet thrombi at the site of the erosional infarct wall coated with dense inflammatory cells (Figure 4C). It was common to observe the formation of platelet thrombi adjacent to the infarct wall (Figures 4D,E), and dense inflammatory cells at the interface of the thrombus (Figures 4D,F) were common. There was evidence for the rapid formation, in the LV chamber, of a large blood clot embedded with platelet thrombi within the clot (Figure 4G). These findings imply that, in hearts with acute MI, the formation of LVT could be due to diverse pathologies that are related to CR, wall erosion, or regional thrombo-inflammatory activation (Figure 4H).

### Incidence and Time-Course of Cardiac Rupture and Left Ventricular Thrombus in the Patient Cohort

**FIGURE 5:**
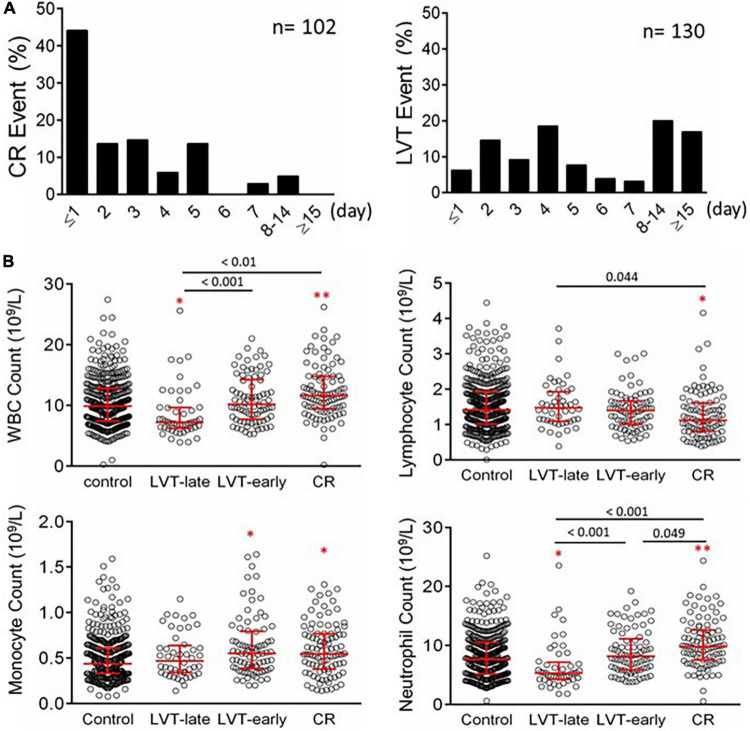
Timing of the onset of CR or echocardiography-based diagnosis of LVT in patients with acute MI **(A)** and counts of peripheral blood inflammatory cells in patients with acute MI without or with LVT or CR **(B)**. The control group consisted of 436 cases with MI without CR or LVT. Patients with LVT were divided into LVT-early (≤ 7 days after MI, *n* = 82) or LVT-late (after day 7 after MI, *n* = 48). The results are medians [interquartile range (Q25, Q75)]. *P < 0.05, **P < 0.01 compared to the control group.

From January 2015 to December 2020, a total of 8,963 patients with acute MI were consecutively admitted to the Department of Cardiovascular Medicine of the First Affiliated Hospital, Xi’an Jiaotong University. CR was diagnosed in 102 patients (1.14%), including a free wall rupture (FWR) in 62 cases, a ventricular septum rupture (VSR) in 33 cases, and a papillary muscles rupture (PMR) in four cases. A combination of FWR and VSR was found in three cases. In terms of the time interval between MI and CR, 59 cases (57.8%) occurred within 48 h (26 cases with CR occurred prior to admission) and 38 cases (37.2%) within 3–7 days (Figure 5A). Nine patients (8.82%) with non-ST-elevation MI (NSTEMI) developed CR. CR occurred in 27 patients who received PCI therapy (primary PCI in 12 cases).

Of the cohort, 130 patients were diagnosed with LVT, representing an incidence of 1.45%. The infarct location of LVT cases was most commonly at the anterior wall (95.4%, 124/130). The time interval between MI and LVT detection ranged from 24 h to over 2 weeks (Figure 5A), with LVT occurring within 7 days in 82 cases (63.1%).

There were 180 in-hospital deaths (2.01%, 180/8,963). The in-hospital mortality for patients with CR was 57.8% (59/102), accounting for 32.8% (59/180) of total in-hospital mortality, only next to acute pump failure (a combination of cardiogenic shock and heart failure, 61.1%, 110/180) but higher than that of malignant arrhythmias (9.4%, 17/180). In-hospital deaths for the four sub-groups are presented in Table 2.

**TABLE 2:** Baseline clinical characteristics at admission in patients with acute MI and without (control) or complicated with cardiac rupture (CR) or left ventricular thrombus (LVT).

Variable	Control	LVT-late	LVT-early	CR	*P*-value
N=	436	48	82	102	
Sex (female, %)	79 (18.1)	7 (14.6)	13 (15.9)	33 (32.4)^a^	0.006
Age (year)	60.1 ± 11.6	59.8 ± 13.7	59.8 ± 13.0	69.7 ± 9.0^a,b,c^	< 0.001
Smoking (n, %)	260 (59.6)	33 (68.8)	50 (61.0)	32 (31.4)^a,b,c^	< 0.001
Alcohol consumption (n, %)	70 (16.1)	14 (29.2)	25 (30.5)^a^	9 (8.8) ^b,c^	< 0.001
Hypertension (n, %)	181 (41.5)	21 (43.8)	38 (46.3)	52 (51.0)	0.351
Diabetes (n, %)	98 (22.5)	8 (16.7)	21 (25.6)	19 (18.6)	0.541
Previous MI (n, %)	31 (7.1)	3 (6.3)	5 (6.1)	3 (2.9)	0.498
Anterior MI (n, %)	201 (46.1)	44 (91.7)^a^	80 (97.6)^a^	56 (54.9)^b,c^	< 0.001
Killip Class III-IV (n, %)	24 (5.5)	8 (16.7)^a^	11 (13.4)	32 (31.4)^a,c^	< 0.001
In-hospital death (n, %)	4 (0.9)	2 (4.2)^a^	1 (1.2)	59 (57.2)^a,b,c^	< 0.001
Cardiac surgery and/or MCS (n, %)	0 (0)	2 (4.2)	0 (0)	21 (20.6)^a,c^	< 0.001
Pericardiocentesis (n, %)	0 (0)	0 (0)	1 (1.2)	16 (15.7)^a,b,c^	< 0.001
Catecholamine use (n, %)	4 (0.9)	2 (4.2)	1 (1.2)	62 (60.8)^a,b,c^	< 0.001
Symptom-to-admission interval >12 h (n, %)	202 (46.3)	14 (29.2)	39 (47.6)	59 (57.8)^b^	0.012
SBP (mmHg)	127 ± 24	121 ± 19	126 ± 21	108 ± 31 ^a,b,c^	< 0.001
DBP (mmHg)	79 ± 16	78 ± 16	82 ± 13	73 ± 20 ^a,c^	0.002
Heart rate (beats/min)	78 ± 16	88 ± 19^a^	88 ± 16^a^	85 ± 25^a^	< 0.001
PCI (n, %)	393 (90.1)	34 (70.8)^a^	73 (89.0)	38 (37.3) ^a,b,c^	< 0.001
LV aneurysm (n, %)	23 (5.5)	26 (54.2)^a^	40 (48.8)^a^	21 (20.8) ^a,b,c^	< 0.001
Hydropericardium (n, %)	375 (9.6)	16 (33.3)^a^	30 (36.6)^a^	17 (16.8)^c^	< 0.001
Platelet count (10^9^/L)	203 [166,241]	243 [198,277]^a^	203 [168,246]^b^	176 [146,214]^a,b,c^	< 0.001
Mean platelet volume (fl)	11.2 [10.4,12.1]	10.9 [9.9,11.7]	11.0 10.0,12.0]	11.5 [10.7,12.6]^b^	0.028
hsCRP ≥ 9.5 mg/L (%)	29.2	54.3^a^	64.6^a^	66.7^a^	< 0.001
Fibrinogen (mg/L)	3.12 [2.53,4.06]	4.01 [2.93,5.35]^a^	4.20 [3.11,5.47]^a^	3.68 [2.86,4.91]^a^	< 0.001
Fibrinogen degradation products (mg/L)	1.51 [1.09,2.42]	3.90 [2.50,6.92]^a^	2.42 [1.57,5.60]^a^	3.11 [1.97,6.77]^a^	< 0.001
D dimer (mg/L)	0.60 [0.38,1.00]	1.65 [0.90,3.20]^a^	1.00 [0.68,2.05]^a^	1.30 [0.74,2.60]^a^	< 0.001

*Data are mean (SD), n (%), or median (interquartile range, IQR).*

*LVT-late, symptom-to-LVT interval > 1 week; LVT-early, symptom-to-LVT interval ≤ 1 week; SBP and DBP, systolic or diastolic blood pressure; MCS, mechanical circulatory support; hsCRP, high-sensitive C-reactive protein; P-values were derived from among-group comparison by the Chi-square test or the Kruskal–Wallis one-way ANOVA. Statistical significance between two groups is indicated as follows: a, vs. control; b, vs. LVT-late; c, vs. LVT- early.*

### Clinical Characteristics of Patients With Cardiac Rupture and Early or Late Left Ventricular Thrombus

As 95% of CR occurred within the first 7 days, patients with LVT were then divided into LVT-early (≤ 7 days) and LVT-late (> 7 days) subgroups based on the consideration that the link with CR would be more evident in LVT-early cases. The baseline characteristics of all four groups of patients are given in Table 2. Compared with the control group, CR patients were older, had a higher female gender ratio, and had lower smoking or alcohol consumption rate. The majority of CR patients were not treated with PCI (62.7%), had a higher Killip class, and had lower blood pressure at admission.

There was no significant difference between the LVT-early and LVT-late groups in any of these parameters. The majority of clinical variables were comparable among LVT subgroups and control groups, except that patients with LVT had higher percentages of anterior localization of MI, a ventricular aneurysm, or hydropericardium (Table 2). Some differences were noticeable between LVT subgroups and CR groups (Table 2). The proportion of anterior MI or PCI therapy rate was lower in CR than in the LVT-late and LVT-early groups. The chance of coexistence with a ventricular aneurysm or pericardial effusion was higher in the LVT subgroups than in the CR group. The percentage of patients with a symptom-to-admission interval > 12 h was higher in the CR vs. LVT-late group. Also, patients with CR were more likely to undergo cardiac surgery, mechanical circulatory assistance, or both.

### Laboratory-Derived Parameters on Inflammation and Cardiac Injury in Patients With Left Ventricular Thrombus or Cardiac Rupture

Blood cell counts were determined at admission and compared among the four groups (Figure 5B). Compared with the control or LVT-late group, patients with CR showed significantly higher WBC, monocytes, and neutrophils but a decreased lymphocyte count. There was no statistical difference between LVT-early and CR with regard to the counts of WBC, lymphocytes, and monocytes, except for a higher neutrophil count in the CR group. The LVT-early group showed significantly higher WBC and neutrophil counts than the LVT-late group. Patients with hsCRP levels greater than the detection limit (9.5 mg/L) were 29% in the control group but 65% and 67% in the CR and LVT-early groups, respectively (both *P* < 0.01, Table 2). The levels of fibrinogen, FDP, and D-dimer were significantly higher in the three event groups than in the control group, but there was no significant difference across the three groups (Table 2). These results imply that both the CR and LVT-early groups exhibited more severe inflammation after MI relative to the control and LVT-late groups.

**FIGURE 6:**
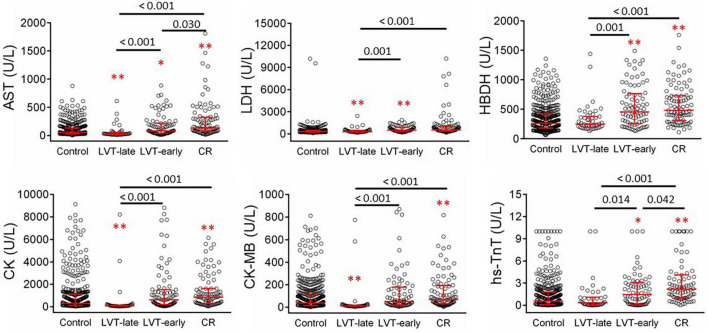
Comparison of serum levels of biomarkers for cardiac injury in patients with acute MI without (control) or with left ventricular thrombus (LVT) or cardiac rupture (CR). Four hundred thirty-six cases were identified in the control groups and 102 cases in the CR group. Patients with LVT were divided into two subgroups of LVT-early (*n* = 82) or LVT-late (*n* = 48). Biomarkers were determined at admission. AST, aspartate transaminase; LDH, lactate dehydrogenase; HBDH, α- hydroxybutyrate dehydrogenase; CK, creatine kinase; CK-MB, creatine kinase-myocardial band; hs-TnT, highly sensitive-troponin-T. *P < 0.05, **P < 0.01 compared to the control group.

Figure 6 shows biochemical markers of cardiac injury among the four groups. Except for LDH, the CR group had significantly higher levels of all markers compared to the control and LVT-late groups. Similarly, the LVT-early group had higher levels of four markers than the control group, while all six markers were higher in the LVT-early group when compared with the LVT-late group. Comparison of these markers among groups revealed an order of CR ≥ LVT-early > LVT-late = Control group. Of platelet parameters, patients with CR had significantly lower platelet counts than the other three groups, together with a higher mean platelet volume (MPV, Table 2). In combination, platelet counts were negatively correlated with MPV (*r* = −0.48, *P* < 0.0001).

### Patients With Dual Complications of Cardiac Rupture and Left Ventricular Thrombus

**FIGURE 7:**
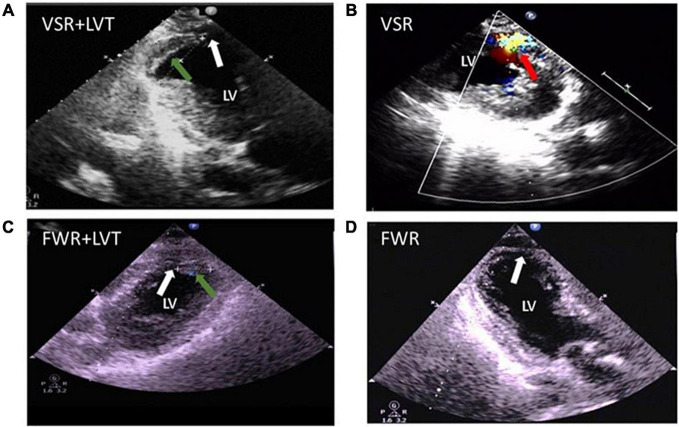
Echocardiographic images from patients complicated with both cardiac rupture (CR) and a left ventricular thrombi (LVT) after acute MI. **(A)** 2-D image (case 1) showing the presence of LVT (green arrow) and defect of the ventricular septum (VSR, white arrow) at echocardiographic examination (on day 14). **(B)** Color Doppler image (case 2) obtained on day 5 after MI revealing the *trans*-septum blood shunt indicating ventricular septum rupture (VSR, red arrow). **(C,D)** 2-D image (case 3 obtained on 5 days) revealed the presence of LVT [green arrow, **(C)**] and the defect of the apical wall [FWR, white arrow, **(D)**].

Three patients in our cohort had both CR and LVT. Their clinical conditions and details of the onset of CR and LVT are presented in Table 3. All three cases experienced MI involving the anterior wall and the presence of a ventricular aneurysm by echocardiography. Pericardial effusion was detected in two cases by echocardiography. Two cases received PCI treatment. VSR was found in two patients on day 1 and day 5 after MI, respectively, and LVT was then detected at the next earliest echocardiographic test (day 14 or day 26, respectively, Figure 7). The remaining case was initially diagnosed with LVT on day 3 and later developed FWR on day 5. Table 3 shows the laboratory parameters of the three cases. Among them, case 2 exhibited very high levels of nearly all cellular and biochemical markers for inflammation or cardiac injury.

**TABLE 3:** Clinical features of 3 patients with acute MI and developed both cardiac rupture (CR) and left ventricular thrombus (LVT).

Variable	Case 1	Case 2	Case 3
Sex	Male	Female	Male
Age (year)	67	78	70
Diagnosis method	Echocardiography	Echocardiography	Echocardiography + Pericardiocentesis
Revascularization	PCI	CAG	PCI
Type of MI	STEMI	STEMI	STEMI
Location of MI	anterior	anterior + inferior	anterior
Rupture type	ventricular septum	ventricular septum	Free wall
LVT localization	Apex	anterior/septum	apex
MI-to-CR interval (day)	1	5	5
MI-to-LVT interval (day)	14	26	3
Ventricular aneurysm (VA)	Yes	Yes	Yes
Timing of VA and LVT	LVT prior to VA	simultaneously	simultaneously
Pericardial effusion	Yes	Yes	No
Killip class	IV	III	I
Percutaneous closure	No	Yes	No
Surgical repair	No	No	No
Days of hospitalization	6	37	5
Outcome	Survived	Deceased	deceased
WBC count (10^9^/L)	7.07	14.73	7.15
Lymphocyte count (10^9^/L)	1.6	1.94	1.41
Monocyte count (10^9^/L)	0.54	0.85	0.59
Neutrophil count (10^9^/L)	4.77	11.9	4.94
Platelet count (10^9^/L)	265	227	178
Mean platelet volume (fl)	9.3	11.7	11.1
Fibrinogen (mg/L)	2.93	5.35	4.71
FDP (mg/L)	5.7	16.85	2.48
D dimer (mg/L)	2.6	5.61	1.53
hsCRP (mg/L)	>9.5	>9.5	n.d.
AST (U/L)	24	282	38
LDH (U/L)	305	1039	413
HBDH (U/L)	290	396	398
Creatine kinase (U/L)	137	135	193
CK-MB (U/L)	11	17	17
hs-TnT (U/L)	0.46	3.7	2.97

*PCI, percutaneous coronary intervention; CAG, coronary artery angiogram only; STEMI, ST-elevation myocardial infarction; hsCRP, highly sensitive C-reactive protein; FDP, fibrinogen degradation products; AST, aspartate transaminase; LDH, lactate dehydrogenase; HBDH, α- hydroxybutyrate dehydrogenase; CK-MB, creatine kinase-myocardial band; hs-TnT, highly sensitive-troponin-T; n.d., not done.*

## Discussion

### Summary of Major Findings

Our joint animal and clinical study was designed to test the potential link between CR and early LVT. We have made a few major discoveries. Experimentally, the presence of IMT and LVT at the site of CR was obtained in all animals with fatal or non-fatal CR using the mouse model of MI with frequent onsets of CR. Histopathological findings strongly support the presence of an inflammo-thrombotic process at the site of CR-LVT. Clinically, while the in-hospital incidence of CR was low, CR contributed to a third of total in-hospital deaths. A majority of patients with CR or LVT experienced it within the first 7 days after MI. In the LVT-early group, the levels of inflammatory cells and biomarkers, as surrogates of cardiac injury, were generally higher than those of the control and LVT-late groups but similar to those of the CR group. Collectively, these findings support our hypothesis that CR and early LVT are mechanistically linked, with CR forming the pathological basis for the development of LVT *via* regional inflammo-thrombotic response.

### Effects of Cardiac Rupture and Left Ventricular Thrombus in a Mouse Myocardial Infarction Model

The mouse is the only laboratory species that, like human patients, develops CR after MI (25, 26). To compensate for the lack of clinical pathology data, the 129sv mouse strain was used. We observed a high incidence of CR in mice that occurs within 2–4 days after MI. A histological examination of mouse hearts with CR identified the presence of severe inflammatory infiltration, intramural hemorrhage, and IMT, which revealed significant findings that are consistent with our previous studies (26, 28). Numerous studies on mice with MI have reported the extent of the inflammation and infarct size as pivotal factors in the pathogenesis of CR (25–27, 34–36). Indeed, interventions that suppress regional inflammation, including the use of anti-platelet therapies, or reperfusion with reduced infarct size are effective in limiting the risk of CR (28, 30, 37). Few studies have examined the pathogenesis of LVT using the murine MI model (28, 29). We also found the presence of acute or chronic LVT in this model. Through a histological examination of CR or non-CR mouse hearts, we revealed for the first time several pathological modes that would be expected to lead to LVT. Among them, rupture or erosion of the infarct wall with regional formation of platelet thrombi is commonly observed. Our histological findings also imply the migration of inflammatory cells from the infarct wall to LVT adjacent to the infarct wall, which would trigger the formation of platelet thrombi. Frantz et al. reported that, in mice with MI, the early onset of LVT (24 h after MI) and interventions that sustained regional inflammation but impaired fibrotic healing increased the incidence of LVT (29). Other studies have shown that interventions that enhance inflammatory monocytes/macrophages together with delayed healing exacerbate ventricular remodeling and the incidence of CR (34, 35, 38).

### The Thrombo-Inflammatory Mechanism in the Formation of Cardiac Rupture and Left Ventricular Thrombus After Myocardial Infarction

We observed in patients with CR complications a reduction in platelet counts and increased MPV, indicating a higher “thrombus burden” and platelet activity (39). In addition to their role in hemostasis and thrombosis, platelets are increasingly recognized as major inflammation-mediating cells in settings of innate or adaptive immunity. Platelets and immune cells interact at the site of disease, thereby contributing to thrombo-inflammatory processes (40). Indeed, our previous studies in mice with acute MI demonstrated platelet-leukocyte interactions that exacerbate inflammatory responses and the onset of CR (25, 28, 30). In the current study, histopathological findings suggest that, following acute MI, thrombo-inflammatory processes, evoked by wall rupture, erosion, or intense inflammatory infiltration, promote both IMT within the infarct myocardium and the formation of LVT. Considering the hemostatic role of IMT that might retard complete and fatal rupture, antiplatelet therapies would be expected to increase the risk of CR. However, we previously observed the opposite, i.e., the use of antiplatelet drugs or platelet depletion in mice almost abolished CR and related IMT, owing to the inhibiting pro-inflammatory action of platelets (28, 30). Clinical studies also indicated a lack of evidence for increment in the risk of CR with the use of anti-platelet drugs (41).

### Clinical Characteristics of Cardiac Rupture and Left Ventricular Thrombus in Patients Post-myocardial Infarction

A previous study showed that approximately 50% of CR cases occurred within 48 h after MI, with 30–40% CR occurring within 24 h (14, 37). We showed a similar time course of CR events, with 25.5% of patients having CR prior to hospitalization. This implies that a fraction of patients with MI with an early onset of CR might have died in the form of sudden death before reaching the hospital. Thus, the true incidence of CR after MI would be higher than the data from hospitalized patients. Such a high proportion of the early onset of CR highlights that CR remains a major challenge to modern cardiology. As reported in the literature, CR is the consequence of infarct expansion, leading to a tear at the weakest location (30, 37). CR is only seen in mice with permanent coronary occlusion, and our previous studies have shown that reperfusion, including delayed reperfusion, abolished the onset of CR (25, 37). Indeed, our patients in the CR group were less likely to receive PCI relative to controls.

We found that the incidence of both CR and LVT after MI was 1.14% or 1.45%, respectively. The figure was markedly lower in earlier clinical reports of 3–10% for CR (8, 15) and 25–40% for LVT (10, 16, 22) but comparable to recent literature (8, 42, 43). We speculate that, under the current management of acute MI, clinical manifestations of CR might have changed in a proportion of patients to the early formation of LVT. In this scenario, the presence of LVT would indicate regional activation of platelet thrombi at the site of non-fatal rupture, hence representing protective hemostasis. Indeed, in the majority of cases, CR and LVT occurred within the first week after MI. It is worth noting that echocardiography would be infrequent in patients without evident indications of LVT, thus delaying LVT detection in our study. Furthermore, despite the fact that the LVT-early and LVT-late groups had comparable clinical characteristics, patients with CR and LVT-early had greater circulating levels of inflammatory cells and cardiac biomarkers. These results indicate that both the CR and LVT-early groups of patients had more severe inflammation and larger infarct size. Our findings are in line with clinical and pre-clinical studies showing that CKMB, hsCRP, and WBC counts are risk factors for CR development (27, 37, 44–46). Recent clinical studies have shown that the neutrophil platelet-lymphocyte ratio was associated with an increased risk of LVT formation (47, 48).

Our finding of the dual presence of LVT and CR in three cases further supports our view of a pathogenetic link between both complications. A similar observation has also been made in previous clinical studies. Domenicucci et al. observed that, among 21 patients with MI and confirmed LVT diagnosis, eight patients (38%) developed CR (16). Compensating for the absence of clinicopathological information, our preclinical study revealed pathological features in the heart of a mouse strain that exhibit a very high risk of developing CR. Interestingly, the presence of LVT or early pathological changes of LVT is observed in the hearts of mice that either died of CR or without a spontaneous CR.

### Potential Implications in the Pathogenesis of Left Ventricular Thrombus

The Triad of Virchow is usually applied in thrombogenesis. Regarding LVT formation following MI, emphasis has been placed on the regional stasis of intra-ventricular blood current as a key factor (49). A few clinical findings in the present study indirectly support our hypothesis of a pathogenetic link between LVT and CR. First, the onset timing of the majority of CR and LVT events was within the first 7 days. Second, both the CR and LVT-early groups had relatively higher levels of inflammatory cell and cardiac biomarkers than in the control and LVT-late groups. Third, a few patients had both LVT and CR. Admittedly, these clinical observations were circumstantial, and the lack of opportunity to study human hearts from deceased patients is a major limitation. To make up for this limitation, we conducted a careful histopathological examination on the hearts of mice without or with a spontaneous CR, and the presence of both CR and LVT in mouse hearts indicates the suitability of this model in addressing our hypothesis. Histopathological findings from the murine model indicate that, for LVT that occurs during the acute phase of MI, one key mechanism is the onset of partial or transmural CR, which results in the formation of IMT. Direct exposure of damaged myocardium to blood in the ventricular chamber could trigger platelet activation, resulting in the formation of IMT and ultimately LVT. Morphological features of IMT strongly indicate a hemostatic action of platelets by blocking the rupture tunnel, albeit this would trigger the formation of LVT following thrombotic growth toward the ventricular chamber, while it was tightly adhered to IMT within the infarct wall. Similarly, we also provided histological evidence for inflammatory cells infiltrating from the infarct wall to the adjacent region of LVT. Takada et al. conducted a pathological study on human hearts from patients with post-acute MI who died suddenly and observed that 44% of 50 cases had an endocardial tear or transmural rupture tracts in the infarct wall, as well as the presence of a mature fresh thrombus at the site of endocardial tears and/or in rupture tracts in the majority of cases, (50) which is equivalent to IMT in the mouse heart with CR.

We observed, in infarcted mouse hearts, several histological modes that could ultimately lead to LVT. Among them, thrombo-inflammation appears to play a key role. A ruptured wall with exposure of necrotic tissues to blood is potent in triggering thrombus formation. An increasing number of studies, including ours, have documented the pro-inflammatory property of platelets in the setting of acute MI (28, 30, 40). Indeed, a blood clot within the LV chamber often contains thrombi-fibrin-rich portion or dense inflammatory cells at the interface with the inflammatory infarct wall, indicating active thrombo-inflammatory processes.

### Study Limitations

Although the mouse MI model is the only one that exhibits CR, there are significant differences relative to the clinical features of CR. This model only simulates FWR owing to infarct localization at the free wall of LV due to the occlusion of the left coronary artery. Furthermore, supra-acute CR (e.g., within 24 h) was seen in 44% of CR cases, while mice with MI developed CR from 48 h to day 5 after MI. The reason for the latter remains to be investigated. Our clinical study was retrospective and observational. An ultrasound system in the vicinity of the cardiology ICU has contributed significantly to this study for the diagnosis of CR (with pericardiocentesis in some cases) and LVT. However, a limitation was the lack of pre-scheduled serial echocardiographic tests, delaying LVT detection by echocardiography. In addition, ultrasound contrast was not used, resulting in some small LVT being overlooked. High-resolution clinical imaging, including the use of contrast in echocardiography and cardiac magnetic resonance (CMR) imaging, would be desirable for a detailed cardiac examination. Considering the difficulty of conducting pathologic (autopsy) verification, CMR imaging, as the current golden standard for LVT confirmation, would compensate for, in large part, such a limitation. Another limitation was the relatively small group size of patients with CR or LVT, which was largely owing to this being a single center-based study. Further research necessitates the establishment of a multi-center and well-planned registry.

### Conclusion and Implications

We have provided preclinical and clinical data that support our hypothesis of an intrinsic link between CR and LVT as significant complications after MI. The coexistence of CR and LVT was evident in mice with acute MI, and similarities in the scale of inflammation and cardiac biomarkers were demonstrated between patients with CR and LVT-early compared to other groups. These findings suggest that the pathogenesis of the early onset of LVT is, at least in part, initiated by endocardial rupture or erosion, which triggers thrombo-inflammatory processes leading to LVT. Future clinical studies involving serial cardiac imaging of patients with MI, would be helpful in validating the potential pathogenetic link between CR and LVT.

## Data Availability Statement

The raw data supporting the conclusions of this article will be made available by the authors, without undue reservation.

## Ethics Statement

The studies involving human participants were reviewed and approved by Ethics Committee of the First Affiliated Hospital of Xi’an Jiaotong University. The written consent form from patients was not obtained owing to the nature of the retrospective study. The animal study was reviewed and approved by Xian Jiaotong University School of Medicine Research Ethics Committee.

## Author Contributions

SM, A-QS, X-LD, X-JD, and QL: experimental design and critical revision of the manuscript. SM, GS, LB, PL, X-JD, and QL: experiments and clinical data collection. SM and X-JD: data analysis. SM, X-JD, and QL: manuscript draft. X-LD, QL, and X-JD: project supervision. All authors contributed to the article and approved the submitted version.

## Conflict of Interest

The authors declare that the research was conducted in the absence of any commercial or financial relationships that could be construed as a potential conflict of interest.

## Publisher’s Note

All claims expressed in this article are solely those of the authors and do not necessarily represent those of their affiliated organizations, or those of the publisher, the editors and the reviewers. Any product that may be evaluated in this article, or claim that may be made by its manufacturer, is not guaranteed or endorsed by the publisher.
